# Self-esteem is associated with loss of autonomy and depression in the elderly

**DOI:** 10.1192/j.eurpsy.2023.1793

**Published:** 2023-07-19

**Authors:** W. Bouali, M. Kacem, W. Haouari, L. Zarrouk

**Affiliations:** 1 Psychiatrie, Faculty of Medicine of Monastir; 2 Psychiatrie, Faculty of Medicine of Sfax, Mahdia, Tunisia

## Abstract

**Introduction:**

Self-esteem is an important aspect of adaptive processes at all ages of life and particularly in older adults: it is linked to the quality of adaptation, well-being, life satisfaction and health.

**Objectives:**

study the links between self-esteem, autonomy and depression in the elderly

**Methods:**

This is a descriptive and analytical study carried out over four months in the offices of three doctors. The population of the study were the consultants whose age was over 65 years the study was made using a pre-established sheet with certain socio-demographic characteristics, the pathological antecedents of subjects. We assessed self-esteem using the Rosenberg scale, autonomy using the activities of daily living scale (KATZ index), and depression using the mini GDS. The analysis of the results was carried out using SPSS 17 software.

**Results:**

Our sample is made up of 54 consultants. The average age was 68 years old. The sex ratio was equal to 0.875. Negative self-esteem, loss of autonomy, depression were observed respectively in 40%, 56.7%, 36.7% of cases. We found an association between negative self-esteem and loss of autonomy (p=0.01) on the one hand, and depression (p<0.0001) on the other.

**Conclusions:**

Our work showed an association between loss of autonomy, depression and negative self-esteem. Depression and negative self-esteem could be a consequence of the loss of autonomy or contributing factors?

**Disclosure of Interest:**

None Declared

